# Long-Term Outcomes and Survival Rates of Patients Undergoing Biopsy Vs. Maximum Safe Resection for Thalamic Lesions: A Short Review on Current Evidence

**DOI:** 10.31661/gmj.v13i.3356

**Published:** 2024-03-01

**Authors:** Ehsan Jangholi, Hadi Anjomshoaa, Parvin Malek Mohammadi, Parisa Rostambeygi, Afsaneh Halili, Mohammad Rahimi, Kamkar Aeinfar

**Affiliations:** ^1^ Brain and Spinal Cord Injury Research Center, Neuroscience Institute, Tehran University of Medical Sciences, Tehran, Iran; ^2^ Department of Neurosurgery, Shariati Hospital, Tehran University of Medical Sciences, Tehran, Iran; ^3^ Department of Psychology and Counselling, Farhangian University, P.O.Box 14665-889, Tehran, Iran; ^4^ Department of Nursing, School of Nursing and Midwifery, Shahid Sadoughi University of Medical Sciences, Yazd, Iran; ^5^ Department of Nursing, Estahban Branch, Islamic Azad University, Estahban, Iran; ^6^ Department of Critical Care Nursing, Isfahan University of Medical Science, Isfahan, Iran; ^7^ Student Research Committee, School of Medicine, Mazandaran University of Medical Sciences, Mazandaran, Iran

**Keywords:** Thalamus, Biopsy, Surgical Resection, Quality of Life, Neurological Deficit

## Abstract

The thalamic lesion is one of the most challenging tumors with significant mortality and morbidities. Current literature highlights the importance of individualized treatment strategies tailored to the specific characteristics of the lesion and the patient. In terms of efficacy, studies have demonstrated that maximal safe resection (MSR) of thalamic lesions can lead to better tumor control, prolonged progression-free survival, and improved overall survival rates compared to biopsy alone. However, the feasibility of achieving MSR is highly dependent on the location, size, and histology of the lesion, as well as the patient’s functional status and overall health. Also, surgical interventions in the thalamus carry inherent risks of neurological deficits, including sensory, motor, and cognitive impairments, depending on the extent of surgical resection and proximity to eloquent neural structures. On the other hand, biopsy remains a valuable diagnostic tool for obtaining tissue samples and establishing a definitive histological diagnosis in cases where MSR is not feasible or poses a high risk of neurological complications. Indeed, biopsy is preferred in patients with advanced age, significant comorbidities, or lesions located in eloquent regions of the thalamus where aggressive surgical resection may result in considerable morbidity. Quality of life (QoL) outcomes, including functional status, symptom burden, and overall well-being, are important endpoints in evaluating the impact of treatment approaches for thalamic lesions on patients’ daily activities. While MSR may offer potential long-term benefits in terms of tumor control and survival outcomes, it may also be associated with a higher risk of neurological deficits and functional impairments that can impact QoL postoperatively. Conversely, biopsy may involve less invasive procedures and shorter recovery times, resulting in better preserved functional status and improved QoL in selected patient populations.

## Introduction

Thalamic lesions present as a challenging clinical scenario due to the critical role of the thalamus in sensory processing, motor control, and cognition [1]. Various etiologies can lead to thalamic lesions, including tumors, vascular malformations, infections, and ischemic events [2][3]. The incidence and prevalence of these lesions vary (e.g., 5.8% reported by Choon et al. [4]), which emphasizes the importance of understanding their impact on patient outcomes and quality of life (QoL).

Indeed, accurate characterization of thalamic lesions is essential for selecting optimal treatment strategies and improving patient prognosis [5]. Advanced imaging modalities, such as magnetic resonance imaging (MRI) or computed tomography (CT) scans, play a crucial role in identifying lesion characteristics and guiding therapeutic decision-making [6][7]. Moreover, the need for precise histopathological diagnosis through procedures like biopsy is vital for personalized treatment planning and prognostication [8].

Also, identifying the long-term outcomes and overall survival (OS) rates associated with thalamus lesions is paramount for optimizing patient care and treatment protocols [9]. Previous studies have explored the impact of different treatment modalities, such as biopsy [10] or maximum safe resection (MSR) [11], on patient outcomes. These studies have highlighted the complexities of managing thalamic lesions, including the risks and benefits of each approach [12].

By consolidating current evidence and incorporating findings from previous studies, healthcare professionals can improve diagnostic accuracy, treatment efficacy, and patient outcomes in thalamic lesion management. Hence, in the current study, we aimed to provide a short review of long-term outcomes and OS rates of patients undergoing biopsy vs. MSR for thalamic lesions.

## Diagnosis and Treatment Planning

Current literature and previous studies emphasize the challenges associated with accurately characterizing thalamic lesions due to their complex anatomical location and diverse etiologies [13][14]. Misdiagnosis and/or inadequate characterization of thalamic lesions can lead to suboptimal treatment outcomes and potential complications [15].

Advanced imaging techniques, such as MRI, CT scan, and positron emission tomography (PET) scan, play a crucial role in the precise diagnosis of thalamic lesions by providing detailed information about lesion location, size, morphology, and surrounding structures [16][17]. These imaging modalities help differentiate various types of thalamic lesions, including tumors, vascular malformations, infections, and ischemic events, accordingly enabling clinicians to tailor treatment approaches [18]. Additionally, incorporating functional imaging modalities, such as functional MRI (fMRI) and diffusion tensor imaging (DTI), can provide valuable information about the functional connectivity of the thalamus and aid in treatment planning [19][20].

Treatment planning for thalamic lesions requires a multidisciplinary approach involving neurosurgeons, neurologists, oncologists, and radiologists to optimize patient care. Previous studies have highlighted the importance of individualized treatment plans based on the specific characteristics of thalamic lesions, the patient’s overall health status, and treatment goals [21][22][23]. For example, while some thalamic lesions may be amenable to surgical resection, others may require targeted therapies, radiation therapy, or symptom management strategies [24].

The integration of precision medicine approaches, such as molecular profiling and genetic testing, is increasingly being explored in the diagnosis and treatment planning of thalamic lesions [25]. By identifying specific molecular markers or genetic alterations associated with thalamic lesions, clinicians can determine treatment strategies, predict treatment response, and optimize patient outcomes [26][27].

## Importance of Determination of Long-Term Outcomes and OS Rate

Nowadays, evidence indicates the importance of evaluating short-term outcomes and long-term OS and functional outcomes in patients with thalamic lesions [28]. In other words, it is necessary for clinicians to make informed decisions regarding treatment options and to counsel patients and their families effectively.

Long-term follow-up studies have revealed that the prognosis of patients with thalamic lesions varies depending on the underlying etiology, lesion characteristics, treatment modalities, and patient-specific factors [9][29][30].

Moreover, assessing long-term functional outcomes in patients with thalamic lesions is crucial for evaluating treatment efficacy, QoL, and rehabilitation needs [31]. Longitudinal studies [32][33] have demonstrated that factors such as lesion location, size, and the extent of surgical resection can impact functional outcomes, including neurological deficits, cognitive impairment, and QoL. Hence, by monitoring these outcomes over time, healthcare providers can tailor rehabilitation programs, supportive care, and interventions to address specific challenges of patients with thalamic lesions [33].

In addition, incorporating patient-reported outcomes and QoL assessments in long-term follow-up studies provides valuable insights into the psychosocial impact of thalamic lesions on patients and their caregivers [34]. These assessments can help identify unmet needs, symptoms, and concerns that may arise over time and inform supportive care strategies to improve overall well-being and patient satisfaction [35].

## Biopsy for Thalamic Lesions

Procedure

Biopsy for thalamic lesions plays a significant role in diagnosing and managing these complex neuroanatomical abnormalities [36]. The procedure involves the minimally invasive collection of tissue samples from the thalamic region using stereotactic techniques guided by advanced imaging modalities such as MRI and/or CT [37][38]. The primary purpose of biopsy is to obtain tissue for histopathological examination to differentiate between various pathologies, including tumors, vascular malformations, infections, and inflammatory conditions [39]. Hence, accurate localization of the biopsy site within the thalamus is essential to minimize risks and maximize diagnostic yield [37]. The procedure of thalamus biopsy is typically performed using the insertion of a biopsy needle or catheter through a small burr hole in the skull under local or general anesthesia [40]. Advanced neuroimaging techniques, such as DTI or neuronavigational systems, may be used to accurately target the lesion without serious damage to surrounding structures during the biopsy procedure [38]. Also, depending on the size and location of the thalamic lesion, different biopsy techniques (such as stereotactic, frameless, or endoscopic approaches) may be employed to ensure safe and effective tissue sampling [41].

Furthermore, current literature highlights the importance of multidisciplinary collaboration involving neurosurgeons, neuroradiologists, neuropathologists, and neuro-oncologists in planning and performing thalamus biopsies [42]. Comprehensive preoperative evaluation, including clinical history, neuroimaging studies, and discussion of risks and benefits, is crucial for optimal patient selection and procedural planning [43]. Post-biopsy management involves close monitoring for potential complications such as hemorrhage, infection, or neurological deficits, with prompt histopathological analysis of the tissue samples to guide further treatment strategies [44][45].

Advantages and Limitations

Biopsy for thalamic lesions offers several advantages and serves as a valuable tool in the diagnosis and management of complex neurological conditions affecting this critical brain region. One of the primary advantages of biopsy for thalamic lesions is its ability to provide a definitive histopathological diagnosis, which is crucial for guiding treatment decisions [46]. Also, it could able to provide treatment strategies, such as surgical resection, radiation therapy, chemotherapy, or targeted therapies, to the specific underlying condition, ultimately improving patient outcomes [47].

Furthermore, thalamus biopsy allows for the molecular characterization of lesions, paving the way for personalized medicine approaches in neuro-oncology and neurology [48]. Advanced molecular profiling techniques, such as next-generation sequencing, can identify specific genetic mutations, biomarkers, or therapeutic targets within thalamic lesions, opening up opportunities for targeted therapies and precision medicine interventions [49][50]. This personalized approach holds promise for improving treatment response rates, minimizing adverse effects, and enhancing overall patient care in the context of thalamic disorders.

On the other hand, thalamus biopsy also presents several limitations and challenges that warrant consideration. One significant limitation is the procedural risks associated with accessing deep-seated thalamic lesions, which may pose technical difficulties and increase the possibility of complications [51]. Careful patient selection, preoperative planning, and vigilant postoperative monitoring are essential to reduce these risks and optimize patient safety during thalamus biopsy procedures [52].

Moreover, the sampling error inherent in biopsy for thalamic lesions can sometimes limit the accuracy of the histopathological diagnosis and subsequent treatment decisions [53]. Due to the heterogeneity of thalamic lesions and the potential for sampling bias, there is a risk of misdiagnosis or incomplete characterization of the underlying pathology based on a single tissue sample. Repeat biopsies or complementary diagnostic modalities, such as advanced neuroimaging, cerebrospinal fluid analysis, or molecular imaging, may be required to enhance diagnostic accuracy and refine treatment strategies in challenging cases [54].

Long-Term Outcomes and Survival Rates After Biopsy

Several retrospective studies [55][56] have reported varying long-term outcomes and survival rates following biopsy for thalamic lesions, depending on the underlying pathology, patient characteristics, and treatment modalities. For instance, in cases of thalamic tumors, such as gliomas, lymphomas, or metastases, survival outcomes have been correlated with factors such as tumor grade, extent of resection, molecular subtypes, and response to adjuvant therapies [57]. In contrast, high-grade gliomas within the thalamus are associated with poorer prognosis and shorter OS than lower-grade tumors or non-neoplastic lesions [58], highlighting the importance of accurate histopathological diagnosis and personalized treatment strategies in optimizing long-term outcomes.

Moreover, studies have demonstrated that the location and size of thalamic lesions can impact long-term OS and functional outcomes following biopsy and treatment. Lesions involving critical thalamic nuclei or white matter tracts may result in significant neurological deficits, cognitive impairment, or disability, influencing patients’ QoL and long-term prognosis [58][59][60].

Additionally, advancements in neuroimaging, neurosurgical techniques, and adjuvant therapies have contributed to improved long-term OS and outcomes for patients undergoing biopsy for thalamic lesions [61]. The integration of stereotactic navigation, intraoperative imaging, neuronavigational, and awake craniotomy approaches has enhanced the precision and safety of thalamus biopsies, minimized the risk of complications, and improved the extent of tumor resection [62]. Furthermore, the development of targeted therapies, immunotherapies, and molecularly guided treatment regimens has expanded treatment options for patients with thalamic tumors, offering new avenues for personalized medicine and improved long-term OS [63].

## MSR

**Figure-1:**
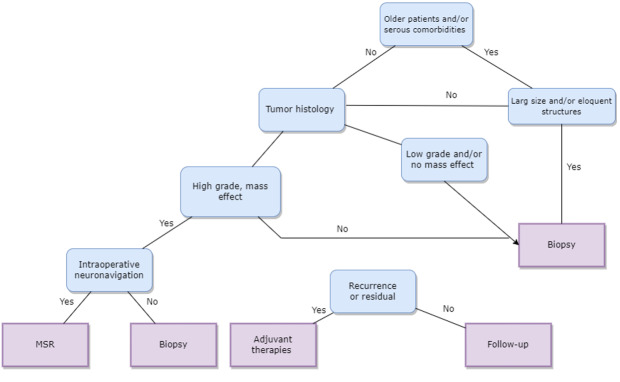
Figure 1. A recommended flowchart for the management of thalamic’ lesions.

Surgical Techniques

The MSR refers to the extent of tumor removal that can be achieved while minimizing the risk of postoperative neurological deficits and preserving vital structures within the thalamus [64]. Surgical planning for thalamic lesions involves a multidisciplinary approach that integrates advanced neuroimaging, functional mapping, and intraoperative monitoring to delineate tumor boundaries, identify eloquent brain regions, and navigate complex anatomical structures [65]. Actually, the goal of MSR is to optimize oncological outcomes by achieving the maximum feasible extent of tumor removal while preserving critical neural pathways and functional domains to minimize the risk of morbidity and optimize patient outcomes [66].

Various surgical techniques have been utilized to facilitate MSR for thalamic lesions, e.g., intraoperative imaging techniques such as intraoperative MRI or CT scans provide real-time feedback to neurosurgeons, enabling them to assess the extent of tumor resection and adjust their surgical approach accordingly to achieve the desired goal of MSR [41][67].

Also, neuronavigational systems enhance surgical precision by providing real-time 3D visualization of tumor margins, adjacent structures, and critical landmarks, guiding the neurosurgeon in achieving MSR while minimizing the risk of postoperative neurological deficits [68].

Advantages and Limitations

One of the primary advantages of MSR for thalamic lesions is the potential for improved oncological outcomes [66]. Indeed, studies have suggested that achieving a greater extent of tumor removal is associated with more prolonged progression-free survival (PFS) and OS rates in patients with thalamic lesions [69][70]. By diligently removing as much tumor mass as safely possible, neurosurgeons aim to reduce the likelihood of tumor recurrence and improve patient outcomes in the long term. Furthermore, maximal tumor resection can help alleviate mass effect-related symptoms, such as intracranial pressure elevation, leading to better symptomatic relief and QoL for patients [71].

Another significant advantage of MSR is the potential to spare critical neurological functions within the thalamus. By utilizing advanced neuroimaging techniques, intraoperative monitoring, and functional mapping, neurosurgeons can identify and preserve essential sensory, motor, and cognitive pathways within the thalamus while removing the tumor [72].

However, MSR for thalamic lesions presents certain limitations and challenges. One of the primary limitations is the risk of damaging critical neural structures during surgery, which can result in postoperative neurological deficits, such as sensory or motor impairments, speech difficulties, or cognitive changes [73]. Balancing the imperative to achieve maximal tumor removal with the need to preserve vital brain regions requires precise surgical planning, expertise, and intraoperative decision-making to minimize the risk of complications and optimize patient outcomes [74]. Furthermore, the location of thalamic lesions can pose technical challenges for achieving MSR, mainly when lesions are centrally located or involve deep structures within the thalamus [75]. Accessing and navigating these regions safely can be complex and may necessitate innovative surgical approaches, such as endoscopic or minimally invasive techniques, to optimize the chances of successful tumor removal while minimizing the risk of surgical morbidity [76].

Long-Term Outcomes and Survival Rates After MSR

The thalamus, a deep-seated and functionally diverse brain structure, poses unique challenges for surgical resection due to its intricate anatomical connections and proximity to vital neural pathways [77]. Despite these challenges, previous research has suggested that maximal tumor removal can lead to symptomatic relief, tumor control, and potentially improved long-term PFS and OS rates in select cases of thalamic lesions [78].

Studies have shown that successful resection of thalamic lesions, such as tumors or vascular malformations, can result in improved QoL, reduced risk of recurrence, and enhanced OS for patients [79][80]. By navigating the intricate anatomy of the thalamus with precision and employing innovative surgical strategies, neurosurgeons strive to achieve therapeutic efficacy while minimizing the risk of postoperative complications and neurological deficits [81].

Nevertheless, factors such as lesion size, location, histology, and pre-existing neurological deficits can influence treatment outcomes and patient prognosis following thalamic lesion resection [82][83][84].

Factors Influencing Treatment Decisions

The decision-making process regarding the choice between biopsy and MSR for thalamic lesions is multifaceted and influenced by various factors elucidated in current literature and previous studies. Understanding these factors is crucial for neurosurgeons and healthcare providers in developing individualized treatment plans that optimize patient outcomes and QoL in managing thalamic lesions [85].

One of the key factors influencing treatment decisions for thalamic lesions is the location and size of the lesion within the thalamus [86]. For example, Cao et al. [87] revealed that the extent of total and subtotal resection was less when the thalamic tumor infiltrated the cerebral peduncles. Indeed, partial resection or biopsy may be a better choice for cases in which it is difficult to resect the tumor totally or sub-totally intraoperatively [87].

Histological characteristics and tumor biology also play a significant role in treatment decision-making for thalamic lesions [88][89]. Lesions with aggressive histology, high-grade malignancies, or molecular features predicting rapid growth and dissemination may necessitate a more aggressive surgical approach with MSR to achieve optimal tumor control and improve long-term PFS and OS rates [89][90]. Conversely, lesions with indolent histologies or low-grade tumors may be amenable to less extensive interventions like biopsy for diagnostic confirmation and ongoing surveillance [91].

Patient-specific factors, including age, overall health status, functional status, and pre-existing comorbidities (e.g., cardiovascular diseases, diabetes, etc.), are critical considerations in determining the optimal treatment approach for thalamic lesions [92][93][94]. Older patients or those with significant medical comorbidities may not tolerate extensive surgical procedures like MSR may benefit more from a less invasive approach, e.g., biopsy, to obtain diagnostic information and guide further management [93]. Conversely, younger and healthier patients with good functional status may be candidates for aggressive surgical interventions to achieve maximal tumor resection and optimize long-term outcomes [95].

Neuroimaging characteristics, such as lesion morphology, extent of mass effect, surrounding edema, and proximity to eloquent structures, also factor into treatment decision-making for thalamic lesions [96]. Advanced imaging modalities, including functional MRI, DTI, and intraoperative neuronavigation, provide valuable information for surgical planning and determining the feasibility of MSR while preserving critical neural pathways [97].

Current Guidelines for Thalamic Lesion Management

Based on current literature and previous studies, a multidisciplinary approach involving neurosurgeons, neurologists, radiation oncologists, and other specialists is recommended to optimize outcomes and individualize treatment strategies for thalamic lesions [98]. In Figure-1, we suggested a simple but informative approach for the management of thalamic lesions. The management of thalamic lesions typically begins with a comprehensive evaluation, including detailed neuroimaging studies such as MRI and possibly functional imaging modalities like DTI and fMRI [99]. Consequently, it could characterize the size, location, and relationship of the lesion to critical neural structures within the thalamus, guiding treatment planning and decision-making [100].

Surgical intervention, either biopsy or MSR, is indicated based on factors such as lesion characteristics, patient age, functional status, comorbidities, and treatment goals [101]. Indeed, MSR is generally preferred for thalamic lesions that are accessible and non-eloquent, with the aim of achieving maximal tumor control while preserving neurological function [74]. In contrast, biopsy may considered for lesions in critical or eloquent areas of the thalamus, cases where the risks of surgery outweigh the benefits of resection, or for diagnostic purposes in lesions with uncertain pathology [93].

For lesions that are not amenable to surgical resection, and in cases of recurrence or residual disease following surgery, adjuvant therapies (such as radiation therapy, chemotherapy, targeted therapies, etc.) may recommended [102][103]. The choice of adjuvant treatment modalities is influenced by factors, e.g., the histology of the lesion, molecular markers, patient-specific characteristics, and treatment goals [104].

Also, in cases where surgical intervention is not feasible or appropriate, a palliative approach focusing on symptom management, supportive care, and improving QoL may be implemented [105]. Multidisciplinary teams, including palliative care specialists, pain management experts, and social workers can provide holistic support for patients with thalamic lesions and their families, addressing physical, emotional, and psychosocial needs throughout the disease course [106][107]. Overall, regarding current studies, we recommended the management of thalamic lesions in some steps as follows:

1. Clinical Assessment:

- Obtain a detailed history and perform a thorough neurological examination.

- Consider the presenting symptoms such as motor deficits, sensory abnormalities, cognitive impairments, and any associated signs.

2. Neuroimaging:

- Utilize MRI scans with contrast to visualize the thalamic lesion and its characteristics.

- Assess the location, size, enhancement pattern, and surrounding structures.

3. Biopsy vs. MSR:

- Determine the need for a biopsy to confirm diagnosis and guide further management.

- Consider the potential benefits of surgical resection for lesions amenable to safe removal.

4. Multidisciplinary Team Discussion:

- Consult with neurosurgeons, neuro-oncologists, neuroradiologists, and neuropathologists to comprehensively evaluate treatment options.

5. Treatment Options:

- Consider treatment modalities such as surgery, radiation therapy, chemotherapy, or a combination based on the type of thalamic lesion.

6. Monitoring and Follow-up:

- Establish a follow-up schedule to monitor treatment response, neurological status, and possible complications.

- Perform periodic imaging to assess for recurrence or treatment-related changes.

7. Symptom Management:

- Address symptoms such as pain, seizures, cognitive deficits, and motor impairments through medications, physical therapy, and supportive care.

8. Rehabilitation and Support:

- Offer rehabilitation services to improve functional outcomes and QoL post-treatment.

- Provide psychological support for patients and their families to cope with the emotional impact of thalamic lesions.

9. Long-Term Monitoring:

- Continuously monitor for long-term effects of treatment, recurrence of lesions, and overall neurological status.

- Adjust the management plan as needed based on the patient’s response and disease progression.

## Future Directions

Future directions and areas for further research in thalamic lesion management hold promise for advancing our understanding and improving treatment outcomes for patients with these complex neurological conditions. Regarding previous studies, several key areas warrant exploration and investigation to enhance the precision and effectiveness of thalamic lesion management strategies. A critical area for further research involves advancing our knowledge of thalamic lesions’ molecular and genetic underpinnings to identify novel therapeutic targets and develop targeted therapies [108]. Understanding the molecular pathways involved in thalamic lesion development, progression, and response to treatment could pave the way for personalized and precision medicine approaches that tailor interventions based on the specific molecular profile of the lesion and the individual patient. Another crucial area for future research is the refinement of imaging modalities and techniques for accurate diagnosis, characterization, and monitoring of thalamic lesions. Ongoing advancements in neuroimaging, such as advanced MRI sequences, PET imaging, and molecular imaging probes, can enhance our ability to non-invasively assess thalamic lesions, characterize their biological features, and monitor treatment response over time [108][109]. Exploring innovative treatment modalities for thalamic lesions, including targeted drug therapies, immunotherapies, gene therapies, and minimally invasive surgical techniques, represents a promising avenue for future research [110].

Investigating the efficacy and safety of emerging treatment approaches in preclinical models and clinical trials could offer new therapeutic options for patients with thalamic lesions, particularly those with challenging-to-treat or recurrent lesions. Furthermore, investigating the role of multidisciplinary care models and integrated supportive services in optimizing outcomes for patients with thalamic lesions is essential. Research focusing on the impact of comprehensive care pathways, including neuro-rehabilitation, palliative care, psychological support, and caregiver education, could provide valuable insights into holistic approaches that address the multifaceted needs of patients with thalamic lesions and improve their overall QoL.

## Conclusion

Previous evidence provides the efficacy, safety, and outcomes of biopsy and MSR for thalamic lesions. However, understanding the benefits and limitations of each approach is crucial for personalized treatment planning and improved patient outcomes. Indeed, implications for clinical practice include the need for a collaborative, evidence-based approach involving a multidisciplinary team and shared decision-making with patients. Hence, updated information about current guidelines, technological advancements, and research findings are essential for providing high-quality care in the management of thalamic lesions.

## Conflict of Interest

The authors declare that the research was conducted without any commercial or financial relationships that could be construed as a potential conflict of interest. Also, one of the authors of the article (E.J) is the deputy editor of the journal. Based on the journal policy, he was completely excluded from any review process of this article, as well as the final decision.
